# From mono- to multi-cellular *in vitro* models: reconstructing colorectal cancer complexity for translational and personalized applications

**DOI:** 10.3389/fcell.2026.1777400

**Published:** 2026-03-16

**Authors:** Gareth Owain Edwards, Francesco Saverio Li Causi, Giorgio Castagnola, Shailendra Singh, Pier Giorgio Amendola

**Affiliations:** 1 Cellomatics Biosciences Ltd., Nottingham, United Kingdom; 2 Department of Medical Surgical Science and Translational Medicine, Sant’Andrea Hospital, Sapienza University of Rome, Rome, Italy; 3 R&D Pharmacon, Life Sciences Consultancy, Sutri, Italy

**Keywords:** co-culture models, colorectal cancer, organ-on- a-chip, patient derived organoids, precision oncology, tumour microenvironment (TME)

## Abstract

Colorectal cancer (CRC) is a heterogeneous disease shaped by diverse molecular subtypes, tumour microenvironmental context, and rapid adaptation under therapeutic pressure. Treatment outcomes remain highly variable, despite advances in screening, molecular profiling, and targeted and immune-based therapies. This is particularly significant in microsatellite-stable disease, where immune exclusion and resistance mechanisms limit therapeutic efficacy. Disease complexity is only partially captured by current biomarkers, contributing to late-stage failure in drug development and suboptimal patient stratification in the clinic. This Mini Review discusses how advanced *in vitro* models (three-dimensional patient-derived organoids, heterotypic co-cultures, organ-on-chip platforms, and *ex vivo* tissue models) accurately reflect CRC pathophysiology and support decision-making in both translational research and precision oncology. These approaches preserve key features of CRC (including tumour architecture, clonal diversity, stromal and immune crosstalk, diffusion barriers, and exposure dynamics), facilitating accurate mechanistic studies, rational combination testing, and functional drug-response profiling. Integrating fit-for-purpose *in vitro* systems into translational workflows can help de-risk therapeutic development and support more personalized, effective treatment strategies for CRC patients.

## 1 Introduction

Colorectal cancer (CRC) presents a major global health burden, with ∼1.93 million new diagnoses and ∼0.90 million deaths in 2022, projected to rise to ∼3.2 million cases and 1.6 million deaths by 2040 due to ageing, population growth, and lifestyle factors (Bray et al., 2024; Morgan et al., 2023; World Health Organization, 2023). While mortality has declined in many populations through screening and multidisciplinary care, incidence trends are shifting. Early-onset CRC (EOCRC, <50 years) has increased in Western nations and parts of East Asia, often presenting at advanced stages with aggressive clinical and molecular features, highlighting the need for tailored prevention, detection, and treatment strategies for younger patients (Araghi et al., 2019; Siegel et al., 2017; Siegel et al., 2019).

CRC arises through interconnected pathways, including the adenoma-carcinoma sequence, serrated neoplasia, and inflammation-associated dysplasia, underpinned by characteristic molecular programmes (Ahmad et al., 2021; Malki et al., 2021; Stefani et al., 2021; Tyagi et al., 2020). Risk is modulated by lifestyle factors such as obesity, physical inactivity, alcohol consumption, tobacco use, diets high in red or processed meat, and chronic inflammation (Hossain et al., 2022; Roshandel et al., 2024). In inflammatory bowel disease, cumulative inflammatory burden maintains elevated CRC risk despite ongoing surveillance (Olén et al., 2020; Shah and Itzkowitz, 2022).

The gut microbiome provides a mechanistic link between diet, inflammation, and carcinogenesis, and may offer biomarkers for early detection and risk stratification (Hofseth et al., 2020; Qu et al., 2023). Family history and hereditary conditions, including Lynch syndrome, familial adenomatous polyposis (FAP) driven by adenomatous polyposis coli (APC) mutations, MUTYH-associated polyposis (MAP), and POLE/POLD1 proofreading defects, require tailored surveillance and management (Hodan et al., 2024; Zaffaroni et al., 2024). Sex-specific differences in tumour biology, incidence, and outcome further highlight gender as a key determinant in prevention and therapeutic strategy (Abancens et al., 2020).

Population-based screening, increasingly recommended from age 45, utilizes fecal immunochemical testing, colonoscopy, flexible sigmoidoscopy, multi-target stool DNA, and emerging AI-assisted polyp detection. These approaches reduce incidence and mortality through removal of premalignant lesions and stage migration, though uptake remains heterogeneous by geography, insurance coverage, and socioeconomic status (Davidson et al., 2021; Gini et al., 2020; Joseph et al., 2020; Lin et al., 2021; Mitsala et al., 2021; Repici et al., 2020; Sánchez-Peralta et al., 2020).

The tumour microenvironment (TME) provides the physiological context for cancer cells, modulating invasion, metastasis, immune escape, and treatment responsiveness. Single-cell and spatial profiling of CRC, including liver metastases, identifies immunosuppressive, metabolically active macrophage populations and spatial TME niches associated with modifying therapeutic outcomes (Larionova et al., 2020; Wang et al., 2021; Wu et al., 2022). Consensus Molecular Subtypes (CMS) classifies CRC into four subtypes: CMS1 (MSI-immune, ∼15%), CMS2 (canonical, ∼40%), CMS3 (metabolic, ∼13%), and CMS4 (mesenchymal, ∼22%), with proportions varying across cohorts (Guinney et al., 2015; Kantha et al., 2025). While not yet routine for treatment selection, CMS classification bridges genotype and microenvironment, forecasting differential therapeutic responses (Guinney et al., 2015; Kantha et al., 2025).

Despite these advances, major barriers still fuel therapeutic resistance: (i) marked biological heterogeneity with rapid, often polyclonal, adaptation under treatment pressure; (ii) largely immune-excluded microsatellite-stable (MSS) disease that limits checkpoint inhibitor efficacy; (iii) a biomarker landscape that is expanding but remains imperfect beyond microsatellite instability/mismatch repair deficiency (MSI/dMMR), human epidermal growth factor receptor 2 (HER2) amplification, and RAS/RAF alterations; and (iv) translational attrition driven by preclinical models that insufficiently capture tissue architecture, clonal diversity, and tumour–microenvironment crosstalk (Bejarano et al., 2021; Durinikova et al., 2021; Guven et al., 2024; Oh et al., 2025; Parseghian et al., 2023; Zhang et al., 2025).

Addressing these gaps requires advanced experimental models and a move from monocellular to multicellular systems to encompass the full range of cell types within the TME and how they communicate to accurately characterize phenotypic responses. This Mini Review focuses on applying three-dimensional (3D) and microphysiological *in vitro* systems, integrated within translational workflows, to improve prediction, map resistance mechanisms, and de-risk combination therapies, thereby enhancing understanding and treatment of this highly complex disease.

## 2 Clinical and molecular overview and current treatments in CRC

Clinicopathological classification in CRC is commonly based on the Tumour-Node-Metastasis (TNM) staging system of the American Joint Committee on Cancer/Union for International Cancer Control (AJCC/UICC), with anatomical site and laterality adding predictive weight. Right-sided tumours are enriched for MSI-high, BRAF^V600E, and serrated-pathway biology and generally have poorer outcomes; left-sided tumours more commonly show chromosomal instability and stronger epidermal growth factor receptor (EGFR)-axis dependence, partly explaining differential responses to EGFR-targeting antibodies (Loree and Kopetz, 2017; Tejpar et al., 2017). Focused molecular characterization, including RAS, BRAF^V600E mutations, MSI/MMR status, and HER2 amplification/overexpression, is now standard, typically via next-generation sequencing, with complementary immunohistochemistry or *in situ* hybridization as appropriate (Mosele et al., 2020). These molecular features and microenvironmental contexts drive disease phenotypes that translational models must recapitulate.

- In localized disease, surgery remains foundational for colon cancer; adjuvant fluoropyrimidine–oxaliplatin combinations are standard for stage III and considered for high-risk stage II, balancing recurrence risk with neurotoxicity (Benson et al., 2024). In right-sided localized colon cancer, MSI/MMR status has been correlated with a higher risk of lymph node involvement, with some authors therefore suggesting a tailored surgical approach for these patients (Muttillo et al., 2024). In rectal cancer, total neoadjuvant therapy (TNT) improves systemic control and pathologic response and can permit organ preservation in rigorously selected complete responders (Benson et al., 2020). In MSI-H locally advanced rectal tumours, exclusive immunotherapy with Dostarlimab, has shown very promising results, with complete clinical response rates in some studies (Cercek et al., 2022). These evolving multimodal strategies highlight the need to understand how radio- and chemotherapy remodel stroma and vasculature and how these changes affect drug penetration and immune cell infiltration, necessitating models that preserve or recreate tissue architecture.
- In metastatic colorectal cancer (mCRC), fluoropyrimidine-based chemotherapy is combined with targeted therapies; anti-vascular endothelial growth factor (anti-VEGF) antibodies are commonly used, particularly in RAS-mutant disease, whereas anti-EGFR antibodies are reserved for selected RAS/RAF wild-type patients, predominantly with left-sided tumours (Benson et al., 2024). Trials such as PARADIGM and ctDNA (circulating tumour DNA)-guided hyper-selection strategies illustrate how clinical and liquid-biopsy biomarkers can refine tissue-based genomics to optimize EGFR antibody use (Shitara et al., 2024; Watanabe et al., 2023). Advanced *in vitro* models should therefore assess combination strategies, EGFR-versus VEGF-driven dependencies, and clonal evolution within a spatially defined TME rather than focusing solely on single-agent regimens.

Molecular niches in mCRC reveal pathway- and lineage-specific therapeutic targets critical for model design. MSI-H/deficient MMR tumours (∼5%) achieve durable benefit from checkpoint blockade, whereas MSS/proficient MMR disease remains largely immune-excluded, responding modestly and context-dependently when the TME is pharmacologically primed, for example through VEGF blockade, multi-tyrosine kinase inhibitors (TKIs) or epigenetic modulation (André et al., 2020; André et 2024; Guven et al., 2024; Ho et al., 2022). Oncogenic drivers such as HER2 amplification, BRAF^V600E, and KRAS^G12C exemplify pathway rewiring in colorectal cancer, whereby effective targeting typically requires rational combination strategies rather than single-agent inhibition (Fakih et al., 2023; Raghav et al., 2024; Yaeger et al., 2023; Yoshino et al., 2023). Collectively, these patterns define immune-inflamed versus immune-excluded states and adaptive feedback circuits that advanced *in vitro* systems should dynamically model, modify, and monitor.

As new agents and combinations are developed, they are compared against late-line standards. Late-line therapies such as trifluridine-tipiracil (±bevacizumab) and regorafenib provide modest survival gains and are constrained by adaptive resistance (Grothey et al., 2013; Prager et al., 2023). Serial ctDNA profiling during EGFR- or KRAS-pathway inhibition reveals polyclonal resistance and clonal decay off-therapy, highlighting the need for repeated or sequential treatments (Parseghian et al., 2023; Topham et al., 2023). Single-cell and spatial analyses of CRC liver metastases further describe immunosuppressive, metabolically active myeloid niches that evolve with treatment (Wu et al., 2022). These data underscore the requirement for advanced *in vitro* models capable of continuous assessment of clonal dynamics and microenvironmental changes, rather than endpoint cytotoxicity alone. Models should preserve or recreate the surrounding architecture, contain key cell types, permit diffusion gradients or continuous dosing, and allow ongoing monitoring via imaging or media sampling. Subsequent sections will focus on state-of-the-art *in vitro* models, examining how effectively they address translational needs and where critical gaps remain.

## 3 Innovative *in-vitro* models to study CRC: from two-dimensional monolayers to three-dimensional systems

**FIGURE 1:**
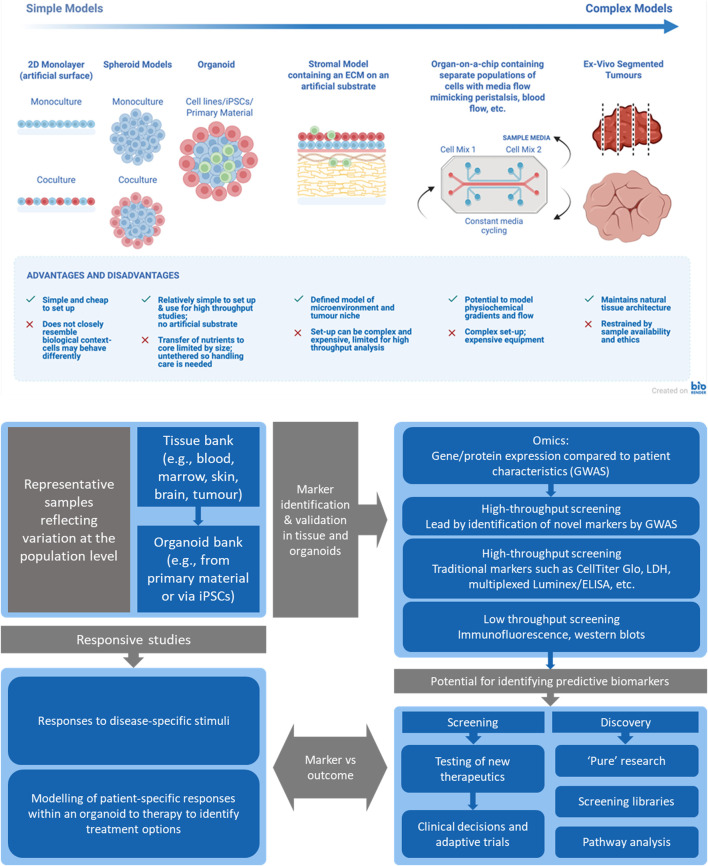
Top) Schematic illustrating the range and increasing complexity of advanced cell culture models, along with the pros and cons of each technique (created using BioRender.com in accordance with its publication license). Bottom) A data-driven translational pipeline—patient → organoid biobank → focused screening/combos → predictive markers → clinical decisions/adaptive trials.

Advanced *in vitro* systems following a hierarchical ladder of increasing complexity have been developed to interrogate CRC biology and guide translational decisions, defining treatment strategies with enhanced efficacy while minimizing off-target effects (Figure 1). Models range from two-dimensional (2D) monolayers to 3D spheroids and patient-derived organoids (PDOs), heterotypic multi-cell co-cultures incorporating stroma and immune cells, perfused organ-on-chip (OoC) devices that recreate pharmacokinetic and physiochemical constraints, and *ex vivo* tissue slices preserving native architecture and drug penetration gradients. Each level addresses specific questions—target validation, drug penetration, resistance circuitry, immune priming—while increasing physiological context and decision-relevant readouts. These systems can be embedded into workflows correlating patient samples, biomarker detection, and downstream responses, informing actionable therapeutic choices in translational medicine.

Classic 2D monolayers remain valuable for reductionist questions and rapid target validation but lack the spatial, mechanical, and cellular complexity governing drug penetration, lineage plasticity, and immune editing in CRC. Clonal drift, matrix-independent signaling, and absence of physiological gradients limit their predictive value. In contrast, 3D systems restore key tumour features, including diffusion barriers, extracellular matrix (ECM)-mediated cues, and inter-patient heterogeneity, producing response profiles closer to *in vivo* function (Yau and Adriani, 2023; Zoetemelk et al., 2019).

CRC PDOs can be established from resections or core biopsies, preserving histological architecture, driver mutations, and microsatellite status (van de Wetering et al., 2015; Yang et al., 2024). Across cohorts, PDO drug responses correlate with matched patient outcomes to standard chemotherapies and targeted agents, complementing genomics for treatment triage and resistance mapping (Ong et al., 2022). Methodological advances—including synthetic hydrogels replacing basement-membrane extracts, air–liquid interface cultures preserving stem-like compartments, and miniaturized high-throughput screening—improve reproducibility and scalability without sacrificing fidelity (Mulero‐Russe and García, 2024; Usui et al., 2018). Living biobanks encompassing multiple disease stages and molecular subtypes enable systematic discovery of biomarkers and combination strategies, providing a robust drug development platform (van Renterghem et al., 2023).

Co-cultures provide controllable, patient-matched systems to test stromal-targeted agents and quantify microenvironmental effects on pharmacodynamics (PD) and potential post-therapeutic relapse (Álvarez-Varela et al., 2022). Organoids generated from primary patient CRC samples have been used to model cancer stem cells (CSCs) which can evade traditional therapies such as 5-Fluorouracil (5-FU) leading to relapse: screening of synthetic retinoid-based compounds targeting CSCs identifies enhanced cytotoxicity and suppressed organoid growth compared to 5-FU (Li S. et al., 2025). Complex systems integrating PDOs with cancer-associated fibroblasts (CAFs), endothelial, and immune cells reintroduce growth-factor exchange, ECM remodeling, and myeloid-driven drug tolerance absent in monocultures. In CRC, CAFs mediate tumour-stromal interactions within the TME (Chen et al., 2024), influencing transcriptional programs and modulating resistance to 5-FU/oxaliplatin through JAK/STAT signaling, thus identifying druggable resistance pathways which may drive advancements in personalized treatment through screening combined treatments in 3D models to overcome chemoresistance mechanisms (Ryu et al., 2024; Zoetemelk et al., 2019). Additionally, mechanistic studies focusing on invasive pathways demonstrate that CRC cells migrate through 3D models in contact with CAFs along elongated protrusions via E-cadherin/integrin-α5β1 engagement with fibronectin fibrils, promoted by the environmental cytokines epidermal growth factor (EGF) and tumour necrosis factor-α (TNF-α) but inhibited by transforming growth factor-β (TGF-β) (Miyazaki et al., 2020).

Organ-on-chips are microfluidic-perfused devices embedding organoids or spheroids in engineered channels to reproduce interstitial flow, oxygen/nutrient gradients, and endothelial barriers. Low-cost “OrganoidChip” platforms maintain CRC PDO viability under flow, enhancing organoid-forming efficiency and enabling medium-throughput testing (Pinho et al., 2021). Drug responses from perfused patient-derived spheroid arrays correlate with *in vivo* outcomes, supporting patient-specific chip-based triage of combinatorial regimens (Ong et al., 2022). Multi-organ constructs, such as gut-liver systems, reproduce first-pass metabolism and microbiome-modulated drug conversion, which is critical for CRC therapy optimization (Vasconez Martinez et al., 2024).

Engineered microenvironments and 3D bioprinting enable fine control over matrix composition, stiffness, and spatial patterning, supporting the generation of more physiologically relevant tumour niches, including vascularized architectures and cancer stem cell–enriched compartments for targeted screening (Zhang et al., 2022). Importantly, bioprinting patient-derived organoids into ordered, standardized microtissues can capture inter-patient variability and reproduce individual drug sensitivities with improved reproducibility and has been reported to support clinical decision-making in selected settings (Chen et al., 2022; Savino et al., 2025; Sbirkov et al., 2021). Together, these advances point to accessible, scalable platforms for disease modelling and drug testing that could unlock new opportunities for personalized therapeutic screening and rational combination selection.

*Ex vivo* tissue slices and micro-organospheres are derived directly from primary tumours, preserving cytoarchitecture, vasculature, and resident immune populations for several days, enabling short-term assessment of drug penetration and immune activation under near-physiologic conditions (Kenerson et al., 2020). CRC slice assays successfully predicted 5-FU ± oxaliplatin responses through histologic and fluorescence readouts, bridging laboratory and clinic (Sönnichsen et al., 2018). Droplet-based “micro-organospheres” generate hundreds of miniaturized replicas from a single specimen, allowing quantitative imaging and machine-learning-assisted response profiling aligned with patient outcomes (Ding et al., 2022). Beyond informing patient-level decisions, these systems support drug development by ranking candidate regimens and stress-testing predictive biomarkers, particularly when integrated with organoid or chip-based models for cross-validation of efficacy, toxicity, and pharmacodynamics (Ding et al., 2022; van Renterghem et al., 2023).

Advanced *in vitro* CRC models are increasingly translating genetic, cellular and microenvironmental complexity into actionable therapy and trial-design hypotheses (Papaccio et al., 2023; van Renterghem et al., 2023). Multi-omics and phosphoproteomics in patient-derived systems link drug response and resistance to defined signaling/metabolic programmes and reveal actionable circuits that can rationalize combinations, for example, in anti-EGFR resistance (Beekhof et al., 2023). In MSS CRC, stromal TGFβ-driven immune exclusion provides a mechanistic rationale for microenvironment-modulating regimens aimed at improving immunotherapy sensitivity (Ho et al., 2022; Tauriello et al., 2018). Looking ahead, the value of these platforms will increasingly depend on longitudinal readouts, such as tracking treatment response, immune modulation and clonal evolution, and on better modelling of long-term adaptive resistance, systemic interactions and multi-organ crosstalk, with further gains expected from standardized microfluidics and integration of multi-omics, computational modelling and liquid biopsy (Ong et al., 2022; Petreus et al., 2021; Yang et al., 2024).

## 4 How advanced models solve concrete problems in drug development and in the clinic

Complex CRC models achieve more than recapitulating morphology: they preserve stromal/immune crosstalk, mimic drug-exposure kinetics, and reproduce spatial/gradient constraints critical in translational workflows. Combined with quantitative readouts, including imaging-based viability, pharmacodynamic biomarkers, phospho-signatures, and ctDNA-aligned markers, these platforms inform two complementary arenas.

### 4.1 In drug development: from mechanism of action (MoA) to PK/PD, regimen, and safety

Goal: reduce late-stage failure by resolving mechanism-of-action, exposure/schedule, and patient safety prior to first-in-human (FIH) trials.

A common hurdle is exposure-response uncertainty for drug combinations identified in 2D screens. A pharmacokinetic-mimetic tumour-on-chip microfluidic system reproduced in vivo–like, time-varying drug exposure in 3D SW620 colorectal cancer spheroids and tested irinotecan (SN38) alone or combined with the ataxia telangiectasia mutated (ATM) inhibitor AZD0156. By integrating spheroid growth/viability with key pharmacodynamic markers, the platform predicted *in vivo* efficacy and enabled upstream optimization of dose and scheduling, reducing reliance on animal studies (Petreus et al., 2021). Modeling payload/partnering for targeted modalities under diffusion barriers is also challenging. Patient-derived xenograft (PDX)-derived organoids showed that a DR5-targeting antibody-drug conjugate (ADC) (Oba01-MMAE) is active in DR5^+^ CRC and synergizes with cyclin-dependent kinase (CDK) inhibition, supporting DR5-based patient selection and combination regimens (Zhou D. et al., 2025).

Assessing safety risks of T-cell engagers is challenging in animal models. A 3D redirect-lysis assay combining colorectal cancer spheroids with healthy adult stem cell-derived organoids showed that cibisatamab, a bispecific antibody targeting carcinoembryonic antigens (CEAs) on cancer cells and CD3 on T cells, induces dose-dependent killing of high-CEA tumour spheroids, while also causing on-target/off-tumour cytotoxicity in healthy organoids with intermediate CEA expression, alongside measurable T-cell activation readouts, supporting pre-FIH dose selection (Anstett et al., 2025). Phosphoproteomic mapping in CRC PDXs coupled with *ex vivo* organoid validation identified druggable resistance targets, informing combination strategies beyond genomics (Beekhof et al., 2023).

### 4.2 In clinical decision-making: from “who benefits” to “what next”

Goal: generate patient-proximal evidence to improve therapeutic decisions.

In immune-excluded MSS disease, the central problem is why checkpoint inhibitors fail and how to convert “cold” tumours. In a genetically engineered mouse CRC model using transplanted tumour organoids in immunocompetent hosts, TGFβ pathway inhibition increased cytotoxic T-cell infiltration and, in advanced metastatic disease, rendered tumours responsive to anti-programmed cell death-1 (PD-1)/programmed death-ligand 1 (PD-L1) therapy, providing a rationale for TGFβ plus checkpoint combinations in MSS CRC (Tauriello et al., 2018). Patient-matched CRC organoid-immune co-cultures identified a tertiary lymphoid structure (TLS) gene signature predictive of anti-PD-1 sensitivity, positioning TLS as a functional biomarker to triage patients (Guo et al., 2025).

When rapid, patient-specific regimen ranking is required, advanced *in vitro* models show predictive success: PDO drug-response profiles across metastatic gastrointestinal (GI) cancers mirrored clinical outcomes with high negative predictive value, enabling pragmatic triage of ineffective options (Vlachogiannis et al., 2018) while precision-cut tumour slices from CRC liver metastases offered a feasible *ex vivo* surrogate to predict drug response (Martin et al., 2019). In MSS CRC, co-culture patient-derived models showed that euchromatic histone-lysine N-methyltransferase 2 (EHMT2) inhibition induces galectin-7, enhances CD8^+^ cytotoxicity, and increases sensitivity to anti-PD-1 therapy *in vivo*, supporting rational combination strategies (Sun et al., 2024). Metastatic lesions can differ functionally from primary tumours, contributing to variable treatment responses in advanced disease (Kjølle et al., 2026). In a prospective, single-centre precision cancer medicine workflow, organoids derived from metastatic biopsies were paired with autologous immune cell cultures for functional testing of standard and immune-based therapies, revealing patient-specific immune modulation of drug response but also a feasibility bottleneck in late-stage patients due to the time required to establish organoids (Kjølle et al., 2026).

Together, these studies illustrate that complex *in vitro* CRC models overcome critical gaps in drug testing, optimizing MoA, exposure, and safety prior to *in-vivo* studies while supplying patient-proximal function to prioritize or de-prioritize therapies in real time. Problem-to-model vignettes are summarized in Table 1, complementing this narrative with actionable takeaways.

**TABLE 1:** Problem-to-model vignettes: actionable takeaways for development and clinical translation.

Unmet clinical/research need	Advanced *in-vitro* model (CRC)	Key mechanistic/functional finding	Clinical/development implication	Representative source(s)
Advancing immunotherapy research and predicting potential safety liabilities in clinical trials (CEA-CD3 bispecifics)	Healthy GI organoids + CRC/GC spheroids co-cultured with allogeneic PBMCs	Dose-dependent killing of CEA^high spheroids; on-target/off-tumour cytotoxicity detectable in CEA^intermediate rectal organoids, minimal in CEA^low small-intestine; robust T-cell activation Assessment of immune checkpoint inhibitors and targets to enhance T-cell toxicity against CRC	Enables preclinical dose selection and safety-risk mitigation for CEA-TCBs prior to FIH Identification of new biomarkers associated with immune cell toxicity against CRC	Anstett et al. (2025)
Effective targeted options for MSS CRC; rational ADC combinations	PDXOs	DR5-targeting ADC (Oba01–MMAE) active in DR5^+^ MSS/MSI-H; synergy with CDK4/6 inhibitors; Cetuximab increases expression of LGR5 which is targeted by novel ADCs	DR5-based selection; supports Oba01 + abemaciclib (ADC + CDK4/6i); combining ADC + existing mAb therapies may enhance efficacy	Zhou D. et al. (2025), High et al. (2025)
PK/schedule optimization (irinotecan/SN38 ± ATM inhibitor) before *in vivo*	PK-mimetic perfused OoC (SW620 Matrigel spheroids)	PK-mimetic exposure reproduces PDX efficacy and yields PD readouts (γH2AX, CC3, Ki-67)	Data-driven regimen/schedule triage upstream of animal/clinical testing; informs PD sampling windows	Petreus et al. (2021), Zhu et al. (2023)
Short-term prediction of FOLFOX benefit with native TME	PCTS from CRC liver metastases	Histology/fluorescence on PCTS predicts response to 5-FU ± oxaliplatin	Predicts 5-FU ± oxaliplatin benefit within days; pragmatic *ex-vivo* surrogate for near-term decisions in mCRC	Martin et al. (2019) Sönnichsen et al. (2018)
Selecting SOC regimen and schedule preclinically	32-plex OoC array seeded with PDX-derived spheroids	Per-patient concordance between on-chip responses and matched PDX efficacy across five SOC regimens; enables testing of schedule/dose under flow	Chip readouts can pre-filter regimens and predict sensitivities to refine exposure profiles before *in-vivo* work	Ong et al. (2022), He et al. (2023)
Functional precision oncology (individual triage)	PDOs from metastatic biopsies	Concordance between PDO drug responses and patient outcomes across regimens	Use PDOs to prioritize regimens for individual patients	Vlachogiannis et al. (2018), Li Y. et al. (2025)
IO priming in MSS-CRC (overcoming cold TME)	PDC-PBMC co-culture	EHMT2 inhibition induces galectin-7, enhances CD8^+^ T-cell cytotoxicity and increases PD-1 responsiveness in MSS-CRC co-cultures	Rationale for combining EHMT2 (G9a) inhibitors with PD-1 blockade in MSS-CRC	Sun et al. (2024), Lv et al. (2024)
Patient-specific microtissue testing	Acoustic bioprinted PDO microtissues or reconstructed stromal tissues	Reproducible microtissues that predict 5-FU response (including in combination with monoclonal antibody-based therapeutics) in individual CRC cases	Supports personalized *ex-vivo* testing workflows and demonstrates bevacizumab enhances 5-FU efficacy	Chen et al. (2022), Takahashi et al. (2024)

Abbreviations: ADC, antibody drug conjugate; CEA-TCB, Carcinoembryonic antigen T-cell bispecific antibody; FIH, First-in-human; GC, gastric cancer; GI, gastrointestinal; OoC, organ-on-chip; PBMC, peripheral blood mononuclear cell; PCTS, Precision-cut tumour slices; PDC, Patient-derived primary tumour cells; PDO, patient-derived organoid; PDX, patient-derived xenograft; PDXOs, PDX-derived organoids; SOC, standard of care; TME, tumour microenvironment.

## 5 Conclusions and future perspectives

Stratification of CRC care is increasingly guided by genotype, anatomy, and microenvironment; however, persistent barriers such as immune exclusion in MSS disease and rapid polyclonal adaptation under targeted therapy continue to limit durable benefit and drive translational attrition (Kennel and Greten, 2025; Parseghian et al., 2023; Pittet et al., 2022; Russo et al., 2021). Advanced *in vitro* systems offer a complementary route to address these gaps. PDOs retain inter-patient heterogeneity and clinically observed drug sensitivities (van de Wetering et al., 2015; Vlachogiannis et al., 2018). Heterotypic co-cultures re-introduce stromal and immune programs that modulate resistance (Álvarez-Varela et al., 2022; Ryu et al., 2024). *Ex vivo* tissue slices preserve native architecture and resident infiltrates (Ding et al., 2022; Sönnichsen et al., 2018) and so accurately reflect the disease in its native environment, whereas organoids are reconstituted from donor material or iPSCs and permit cellular or ECM combinations pertinent to the study design along with mimetic TME to be defined (Strating et al., 2023; Varinelli et al., 2022). Perfused OoC platforms impose pharmacokinetic and transport constraints (Ong et al., 2022; Pinho et al., 2021); whilst the cost is greater than singular PDO-based systems, the ability to define barriers and physiological flows provides a basis for replacement of animal models to model human tissues and multi-organ metastases (Avula and Grodzinski, 2024; Konopka et al., 2026). When applied, these models convert complex biology into actionable decisions by aligning functional readouts with clinical biomarkers and by stress-testing drug combinations and schedules prior to animal work or patient exposure.

Limitations persist, including epithelial bias in tumour-only PDOs, partial immune representation, and cost/throughput constraints in complex OoCs (Antunes et al., 2022; Chen et al., 2022; Cordeiro et al., 2024). From a feasibility and regulatory standpoint, the dominant bottlenecks are (i) time-to-model within late-stage clinical decision windows, (ii) inter-laboratory standardisation and reproducibility (including QC and reference benchmarks), and (iii) validation in a clearly defined context of use that links model readouts to clinically meaningful endpoints. These priorities align with regulatory efforts to advance New Approach Methodologies (NAMs), including recent FDA initiatives encouraging the integration of alternative methods into drug development decision-making (U.S. Food and Drug Administration, 2023; U.S. Food and Drug Administration, 2025a). Accordingly, the practical gating factors for adoption are fit-for-purpose validation, reproducibility across sites, and alignment with qualification/implementation pathways (U.S. Food and Drug Administration, 2025b). Strategies to mitigate these include micro-organospheres, standardized synthetic matrices, living biobanks, and precompetitive consortia that link models with curated clinical registries (Ding et al., 2022; van Renterghem et al., 2023).

Through the development and implementation of advanced models as standardized therapeutic testing platforms, animal replacement models and clinical tools, specific guidance and regulatory frameworks will be required to ensure ethical application and scientific rigour. Regulatory adoption will depend on demonstrating that model-guided decisions improve response rates, prevent disease-mediated organ loss, or overcome resistance mechanisms while maintaining patient safety (Petreus et al., 2021; Zhou L. et al., 2025).

In summary, advanced CRC *in vitro* platforms are evolving beyond mechanistic tools into decision engines that (i) increase predictive accuracy, (ii) identify and pre-empt resistance circuitry, and (iii) rationalize drug combinations and sequencing where conventional systems fail. Embedding fit-for-purpose models with quantitative readouts upstream of trials provides a realistic path to lower phase-II attrition and accelerates the translation of mechanism into medicine for people living with colorectal cancer.

## Glossary

ADC: Antibody-drug conjugate
AI: Artificial intelligence
AJCC/UICC TNM: American Joint Committee on Cancer/Union for International Cancer Control
APC: Adenomatous polyposis coli
ATM: Ataxia telangiectasia mutated
CAF: Cancer-associated fibroblast
CDK: Cyclin-dependent kinase
CEA: Carcinoembryonic antigen
CEA-TCB: Carcinoembryonic antigen T-cell bispecific antibody
CMS: Consensus molecular subtype
CRC: Colorectal cancer
CSC: Cancer stem cell
ctDNA: Circulating tumour DNA
ECM: Extracellular matrix
EGF: Epidermal growth factor
EGFR: Epidermal growth factor receptor
EHMT2: Euchromatic histone-lysine N-methyltransferase 2
ELISA: Enzyme-linked immunosorbent assay
EOCRC: Early-onset colorectal cancer
FAP: Familial adenomatous polyposis
FIH: First-in-human
5-FU: 5-Fluorouracil
GC: Gastric cancer
GI: Gastrointestinal
GWAS: Genome-wide association study
H2AX: Histone H2A.X
HER2: Human epidermal growth factor receptor 2
iPSC: Induced pluripotent stem cell
KRAS: Kirsten rat sarcoma viral oncogene homologue
LDH: Lactate dehydrogenase
mCRC: Metastatic colorectal cancer
dMMR: Deficient mismatch repair
MAP: MUTYH-associated polyposis
MoA: Mechanism of action
MSI: Microsatellite instability
MSI-H: Microsatellite instability-high
MSS: Microsatellite stable
MUTYH: MutY DNA glycosylase
OoC: Organ-on-chip
PBMC: Peripheral blood mononuclear cell
PCTS: Precision cut tumour slice
PD-1: Programmed cell death-1 protein
PD-L1: Programmed death-ligand 1
PDC: Patient derived primary tumour cells
PDO: Patient derived organoid
PDX: Patient derived xenograft
PD: Pharmacodynamic
PK: Pharmacokinetic
POLE/POLD1 DNA: Polymerase epsilon/delta 1
RAS/RAF: Rat sarcoma virus/Rapidly accelerated fibrosarcoma
SOC: Standard of Care
TKI: Tyrosine kinase inhibitor
TLS: Tertiary lymphoid structure
TGFβ: Transforming growth factor-β
TME: Tumour microenvironment
TNFα: Tumour necrosis factor-α
TNT: Total neoadjuvant therapy
VEGF: Vascular endothelial growth factor
